# A Household Cluster of Tick-Borne Encephalitis in Belgium in 2025: Is the Epidemiology Evolving?

**DOI:** 10.3390/v18050491

**Published:** 2026-04-23

**Authors:** Hélène Boogaerts, Janne Tollenaere, Kim Bekelaar, Els Oris, Sarah Resseler, Baptist Declerck, Dorien Van den Bossche, Marjan Van Esbroeck, Deborah Steensels

**Affiliations:** 1 Department Microbiology, Hospital Oost-Limburg, 3600 Genk, Belgium; 2 Department of Neurology, Hospital Oost-Limburg, 3600 Genk, Belgium; 3 Institute of Tropical Medicine, 2000 Antwerp, Belgium

**Keywords:** tick-borne encephalitis, *Ixodes ricinus*, emerging infectious diseases

## Abstract

Despite serological evidence of tick-borne encephalitis virus (TBEV) circulation in Belgian animals since 2007, confirmed autochthonous human infection was only first documented in 2020. We review the current national epidemiologic situation and investigate a household cluster of confirmed autochthonous cases identified in 2025. A cohabiting couple experienced a near-simultaneous onset of meningoencephalitis and tested positive for TBEV-specific IgM and IgG, with confirmation by PRNT90. One patient reported a recent tick bite, and both patients reported consumption of unpasteurized milk and goat cheese, suggesting possible alimentary transmission. The identification of Case 2, who lacked neurological symptoms at presentation and was only tested due to the index case, illustrates the risk of missed diagnoses and supports the notion that human TBEV infection is likely underdiagnosed in Belgium. These findings underscore the need to increase clinical awareness, strengthen surveillance, and reinforce prevention strategies. TBE should be considered in the differential diagnosis of patients presenting with non-specific fever or neurological syndromes such as meningoencephalitis, particularly during the spring-to-autumn tick activity season.

## 1. Introduction

Tick-borne encephalitis (TBE) is a vaccine-preventable viral infection of the central nervous system caused by the tick-borne encephalitis virus (TBEV) which in Europe is transmitted primarily through the bite of infected *Ixodes ricinus* ticks [1]. The disease ranges in severity from mild febrile illness to severe meningoencephalitis, and is increasingly recognized beyond its traditional endemic areas in Central and Eastern Europe [2].

The present article reviews the available literature on the presence of TBEV in Belgium and reports two newly detected autochthonous human TBE cases identified in 2025.

## 2. Materials and Methods

### 2.1. Literature Review and Data Synthesis

A targeted search of the published literature was conducted to synthesize the available information regarding the presence, circulation, and human cases of tick-borne encephalitis virus (TBEV) in Belgium. The review included articles and official reports pertaining to animal seroprevalence, entomological surveillance and human case data.

### 2.2. Case Identification and Clinical Data Collection

Clinical data, including history of tick exposure, travel history, symptom onset, and clinical progression for the two reported 2025 cases, were collected from patient records upon admission and during follow-up. Serology, cranial CT, and cerebrospinal fluid (CSF) analyses were performed as part of the diagnostic workup for both cases.

## 3. Results

### 3.1. Evidence of TBEV Circulation in Belgium

Serological studies in Belgian sentinel animals have demonstrated TBEV-specific antibodies since 2007, indicating long-standing viral circulation. Antibodies have been detected in multiple species, including dogs, cattle, wild boar, sheep, and roe deer, sometimes at high titers [3,4,5]. TBEV seroprevalence in wild boar in Belgium was assessed in two studies, revealing rates of 4.20% in 2013 [3] and 9.27% in 2019–2020 [4]. Although previous studies did not detect TBEV in ticks collected in Belgium [4,6,7], the repeated serological findings in animals, together with circulation in neighboring countries, suggested positivity in ticks in Belgium. This was confirmed in 2024 by the direct detection of TBEV RNA in *Ixodes ricinus* nymphs and adult ticks, providing entomological evidence of virus circulation in Belgium [8].

### 3.2. Human Cases of TBE in Belgium and Surveillance

No autochthonous human TBE cases were reported in Belgium before 2018. Prior to 2012, no structured national TBE surveillance existed in Belgium, and no human cases were formally reported in the period 2000–2011 [9]. Between 2012 and 2017, all identified cases were imported infections, reported through voluntary notification to the National Reference Centre for Arboviruses (Institute of Tropical Medicine, Antwerp) [10]. The epidemiological picture began to change in 2018 with the detection of the first two possibly or probably autochthonous human infections [10,11], followed by the first confirmed locally acquired cases in 2020 [10,12], formally establishing the presence of human TBEV transmission in Belgium. This is consistent with a broader epidemiological trend across Europe: between 2012 and 2020, the number of reported TBE cases increased across the EU/EEA, with a notable northwestward geographic expansion into regions previously considered non-endemic, including neighboring countries of Belgium such as the Netherlands and Germany [2].

Additional autochthonous cases were reported in 2024 (*n* = 2) [10,13] and in 2025 (*n* = 3). In this report, we describe two of the cases reported in 2025, which were diagnosed in our hospital.

**Figure 1:**
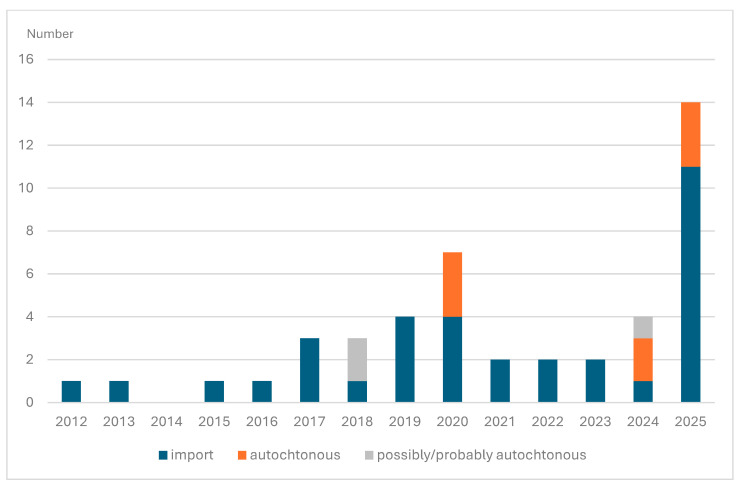
Numbers of TBE cases in Belgium over time. Source: Belgium national reference center for TBE—Institute of Tropical Medicine (2012–2024) [10]; Van Esbroeck, personal communication (2026).

Figure 1 provides an overview of imported, autochthonous, and possibly/probably autochthonous TBE cases reported in Belgium from 2012 through 2025.

Human TBE is likely underdiagnosed due to limited clinical awareness, passive surveillance, and the fact that TBEV is not routinely tested in patients presenting with unexplained neurological symptoms. To improve data collection, the NeuroSurv network was launched in January 2024 [14], and (suspected) autochthonous TBE infections were added to the list of notifiable diseases in Belgium the same year [15].

### 3.3. Two New Autochthonous TBE Cases in Belgium (2025)

Case 1: A 58-year-old female with newly diagnosed hyponatremia (serum sodium 128 mmol/L) was referred. She had a multi-month prodromal history of unexplained anorexia, nausea, weight loss, and recurrent oral candidiasis, with a recent unremarkable gastroenterological workup. In the days preceding admission, she developed a fever (38.3 °C).

Initial laboratory investigations showed mildly elevated C-reactive protein (11.4 mg/L) but otherwise normal inflammatory and hematological parameters. Despite correction of hyponatremia, she developed progressive encephalopathy without focal deficits or meningeal signs. An extensive infectious workup (including blood cultures, urine culture with Legionella antigen, and chest CT) was negative.

Cranial CT was unremarkable. EEG demonstrated generalized slowing consistent with encephalopathy. Cerebrospinal fluid (CSF) analysis revealed lymphocytic pleocytosis (27 cells/mm^3^), elevated protein (72 mg/dL), and elevated lactate (2.5 mmol/L). A repeat lumbar puncture two days later showed progression, with 105 cells/mm^3^ (87% lymphocytes, 13% monocytes/macrophages) and protein of 89 mg/dL. Brain MRI and PET-CT were unremarkable. Further history revealed a recent tick bite [several/around four] weeks before the symptom onset. No erythema migrans was reported.

Case 2: The patient’s husband, a 55-year-old male, had recently been hospitalized in the hematology department with relative leukopenia and similar complaints: fever, malaise, anorexia, and weight loss,. Given the symptomatic overlap, he underwent an elective lumbar puncture, which also demonstrated a mild lymphocytic meningitis. The husband had no recollection of a recent tick bite, but did mention the consumption of unpasteurized milk and goat cheese produced by a local farm in Limburg by him and his partner in the same period of their prodromal symptom onset.

Diagnosis and Outcome: Both patients improved with supportive care (intravenous fluids, antipyretics) and were discharged in good clinical condition after one week.

Given the recent history of a tick bite in patient 1, TBE was added to the differential diagnosis, and serological testing was added for tick-borne encephalitis (TBEV) in both cases. Serum from Case 1 was collected 7 days after the onset of hyponatremia (approximately 20 days after initial non-specific symptoms). Serum from her spouse (Case 2) was collected 4 days later (approximately 26 days after his non-specific symptom onset). Both samples returned positive for TBEV IgM and IgG antibodies. Neither patient had a history of vaccination against TBEV.

Antibody screening was performed at the National Reference Center (NRC) using a mosaic immunofluorescence assay (IFA) (Flavivirus Profile 2, EUROIMMUN AG) [13 => 12]. To confirm specificity, a 90% plaque-reduction neutralization test (PRNT90) was performed. Table 1 provides an overview of the laboratory findings and exposure characteristics of the two confirmed autochthonous TBE cases in Belgium in 2025.

At two-month follow-up, both patients reported persistent post-infectious symptoms, including concentration difficulties, fatigue, and irritability.

## 4. Discussion

### 4.1. Endemicity and Public Health Significance

The detection of confirmed autochthonous TBE cases in Belgium since 2020, including the confirmed cluster of two cases reported here, may reflect an evolving epidemiological situation, and establishes TBEV as a public health concern. Adjadj et al. suggested an increase in TBEV seroprevalence in wild boar in Belgium (4.20% in 2013 and 9.27% in 2019–2020) but stated that it was difficult to ascertain this increase, given the differences between the two studies with regard to sample size, screening method, and the Flemish provinces included [4]. Overall, the long-standing serological evidence in wildlife (since 2007) suggests that the virus has been present in Belgium for over a decade [3,4]. The current increase in reported human cases could likely reflect an increased clinical and public health awareness due to new surveillance measures [10,15]. This is illustrated by Case 2, who would not have been tested for TBEV in the absence of the index diagnosis in Case 1.

Alimentary transmission is a well-established, albeit less reported, route of infection, accounting for an estimated 1% of all human TBE cases, most of which are reported in eastern and central Europe [16].

### 4.2. Diagnostic and Clinical Challenges

The limited number of confirmed TBE cases in Belgium likely represents only the “tip of the iceberg.” Most TBEV infections are asymptomatic or manifest as a mild, self-limiting febrile illness that does not prompt diagnostic testing. In patients who do develop neurological symptoms, TBEV is not routinely included in the diagnostic workup, reflecting limited clinical awareness and contributing to underdiagnosis. The clinical course of Case 1 illustrates these challenges, with a prolonged and non-specific prodromal phase and an initial diagnostic focus on hyponatremia, underscoring how TBE can present atypically and delay diagnosis.

### 4.3. Transmission Route Implications

The concurrent onset of symptoms in a cohabiting couple from the same geographic area is noteworthy and suggests a shared exposure. Although Case 1 reported a recent tick bite and Case 2 did not, both individuals did mention going on frequent walks in the forest. Both individuals also reported consumption of unpasteurized milk bought of a local farmers’ market. The almost concurrent onset of symptoms makes an alimentary transmission route through raw dairy consumption plausible.

The patients in the household cluster in Belgium were unable to recall the exact date of their last consumption of raw milk; however, they reported purchasing and consuming it on multiple occasions in the weeks preceding symptom onset. The median incubation period for alimentary TBEV transmission is 3.5 days [16], but given the prolonged prodromal course, the exact timing and transmission date remains uncertain. The possibility warrants further investigation of local dairy supply chains, and highlights the importance of public awareness regarding the risks associated with consumption of unpasteurized milk products. In the present cases, the competent health authority was notified. To the authors’ knowledge, no on-site investigation of the suspected farm was conducted, possibly representing a missed opportunity to confirm the alimentary transmission route.

Notably, outbreaks linked to alimentary transmission have been documented in non-endemic regions, typically associated with the consumption of unpasteurized dairy products, and often involving clusters of cases exposed to a common source, as illustrated by a large outbreak in France in 2020 linked to raw goat milk, which affected 43 individuals [17]. Investigation of potential alimentary transmission should ideally extend beyond the dairy supply chain to include on-site surveys of the suspected farm, encompassing serological screening of animals and entomological assessment of tick populations, to better characterize the risk of foodborne TBEV exposure.

### 4.4. Conclusions and Recommendations

The increase in reported TBE cases in Belgium and the concern of underdiagnosis mandate increased clinical awareness among physicians, ensuring TBE is included in the differential diagnosis for patients presenting with non-specific fever or neurological syndromes such as meningoencephalitis, particularly during the spring-to-autumn tick activity season.

Furthermore, national surveillance efforts must be strengthened to guide public health interventions, including targeted messaging regarding tick bite prevention and the risks of raw milk consumption.

## Figures and Tables

**Table 1:** Laboratory results and exposure characteristics of the two confirmed autochthonous TBE infections cases in Belgium in 2025.

Case No.	Symptom Onset Date	Symptoms/Presentation	Likely Route, Time	Sample Type, Days After Symptom Onset	Flavivirus IFA	PRNT90 Titer
1	20 June 2025	Nausea, anorexia, weight loss, fever, encephalopathy	Tick bite, (forest, exact time not known (June 2025)), or raw dairy consumption.	Serum; ± 20 days	TBEV IgM+ TBEV IgG+	1:57
2	18 June 2025	Malaise, anorexia, weight loss, lymfocytic meningitis	No known tick bite, frequent walks in the forest, or raw dairy consumption.	Serum; ± 26 days	TBEV IgM+ TBEV IgG+	1/165

IFA—immunofluorescence assay; PRNT90—plaque-reduction neutralization testing at 90% sensitivity; TBEV—tick-borne encephalitis virus. + positive; − negative; ± = approximately.

## Data Availability

The original contributions presented in this study are included in the article. Further inquiries can be directed to the corresponding author.

## Abbreviations

The following abbreviations are used in this manuscript:

TBEV	Tick-borne encephalitis virus
CSF	Cerebrospinal fluid
NRC	National reference center
CT	Computed tomography
PET	Positron emission tomography
